# Nitrogenase MoFe protein from *Clostridium pasteurianum* at 1.08 Å resolution: comparison with the *Azotobacter vinelandii* MoFe protein

**DOI:** 10.1107/S1399004714025243

**Published:** 2015-01-23

**Authors:** Li-Mei Zhang, Christine N. Morrison, Jens T. Kaiser, Douglas C. Rees

**Affiliations:** a Division of Chemistry and Chemical Engineering, California Institute of Technology, Pasadena, CA 91125, USA; b Howard Hughes Medical Institute, California Institute of Technology, Pasadena, CA 91125, USA

**Keywords:** FeMo cofactor, P cluster, metalloproteins, iron–sulfur clusters

## Abstract

The X-ray crystal structure of the nitrogenase MoFe protein from *Clostridium pasteurianum* (Cp1) has been determined at 1.08 Å resolution by multiwavelength anomalous diffraction phasing. Cp1 and the ortholog from *Azotobacter vinelandii* (Av1) represent two distinct families of nitrogenases, differing primarily by a long insertion in the α-subunit and a deletion in the β-subunit of Cp1 relative to Av1. Comparison of these two MoFe protein structures at atomic resolution reveals conserved structural arrangements that are significant to the function of nitrogenase. The FeMo cofactors defining the active sites of the MoFe protein are essentially identical between the two proteins. The surrounding environment is also highly conserved, suggesting that this structural arrangement is crucial for nitrogen reduction. The P clusters are likewise similar, although the surrounding protein and solvent environment is less conserved relative to that of the FeMo cofactor. The P cluster and FeMo cofactor in Av1 and Cp1 are connected through a conserved water tunnel surrounded by similar secondary-structure elements. The long α-subunit insertion loop occludes the presumed Fe protein docking surface on Cp1 with few contacts to the remainder of the protein. This makes it plausible that this loop is repositioned to open up the Fe protein docking surface for complex formation.

## Abstract 2

Determination of the nitrogenase MoFe protein from *C. pasteurianum* at 1.08 Å resolution and comparison to its distinct ortholog from *A. vinelandii* at atomic resolution reveals conserved structural arrangements that are significant to the function of nitrogenase.

## 1. Introduction   

Biological nitrogen fixation is the process of reducing atmos­pheric dinitrogen to a biologically available form, such as ammonia, catalyzed by the enzyme nitrogenase found in certain bacteria and archaea (Burgess & Lowe, 1996 ▶; Howard & Rees, 2006 ▶; Seefeldt *et al.*, 2009 ▶; Hu & Ribbe, 2010 ▶). This reaction is powered by adenosine triphosphate (ATP) hydrolysis, with the requisite electrons supplied from ferredoxin or flavodoxin. Nitrogenase is composed of two metalloprotein components, the molybdenum–iron (MoFe) protein and the iron (Fe) protein in the well characterized molybdenum nitrogenase system. The MoFe protein forms a dimer of heterodimers (α_2_β_2_) containing two complex metalloclusters in each αβ heterodimer: the [8Fe–7S] P cluster bridging the α-subunit and β-subunit and the [7Fe–Mo–9S–C–homocitrate] FeMo cofactor buried inside the α-subunit. The Fe protein is a γ_2_ dimer with one [4Fe–4S] cluster between the two monomers. During nitrogen fixation, the Fe protein forms a transient complex with the MoFe protein and transfers electrons to the MoFe protein with concomitant hydrolysis of ATP. The FeMo cofactor provides the active site for substrate reduction, while the P cluster is believed to serve as a relay for electron transfer from the [4Fe–4S] cluster of the Fe protein to the FeMo cofactor.

While nitrogenase MoFe proteins are highly conserved among the nitrogen-fixing species examined so far, differences do exist. The *Azotobacter vinelandii* MoFe protein (Av1) and the *Clostridium pasteurianum* MoFe protein (Cp1) have been recognized as representatives of two distinct groups: group I and group II, respectively (Wang *et al.*, 1988 ▶; Howard *et al.*, 2013 ▶). Cp1 and Av1 share the lowest sequence identity (∼37%) among the well studied MoFe protein homologs. In particular, the group II MoFe proteins represented by Cp1 have a long insertion in the C-terminus of the α-subunit and a long N-terminal deletion in the β-subunit compared with the group I proteins represented by Av1 (Howard *et al.*, 2013 ▶). Correlated with the structural differences, Cp1 and Av1 show distinct physicochemical properties (such as midpoint redox potentials and optimal pH for enzyme activity; O’Donnell & Smith, 1978 ▶; Kim *et al.*, 1993 ▶). One of the most significant differences is that, in contrast to other MoFe proteins, Cp1 rarely exhibits promiscuity towards orthologous Fe proteins from other bacterial species, while the *C. pasteurianum* Fe protein (Cp2) forms an inactive complex with Av1 (Emerich & Burris, 1978 ▶). Considering these differences between Cp1 and Av1 in sequence, structure and certain properties, the structural arrangements conserved in both proteins are likely to be essential for the nitrogenase activity. Therefore, the identification of the structural motifs that are conserved between Cp1 and Av1 will render insights into the functional mechanism of nitrogenase.

A structure of Cp1 has previously only been reported at 3 Å resolution (PDB entry 1mio; Kim *et al.*, 1993 ▶). While a 2.2 Å resolution Cp1 structure has been determined, the refined structure has not been deposited in the PDB (Bolin *et al.*, 1993 ▶). In contrast, Av1 has been characterized at atomic resolution [1.16 Å resolution (PDB entry 1m1n) and 1.0 Å resolution (PDB entry 3u7q) ; Spatzal *et al.*, 2011 ▶; Einsle *et al.*, 2002 ▶]. Additionally, the *Klebsiella pneumoniae* MoFe protein (Kp1), which is closely related to Av1 with ∼70% sequence identity, has been characterized at 1.6 Å resolution in different oxidation states (PDB entries 1qh1 in the reduced state, 1qh8 in the oxidized state and 1qgu in the mixed oxidation state; Mayer *et al.*, 1999 ▶). We report in this paper the crystal structure of Cp1 determined at 1.08 Å resolution, and describe the three-dimensional structure alignment against the 1.0 Å resolution Av1 structure, with a focus on the structures of the two metalloclusters and their surrounding environment. Unless specified elsewhere, the 1.08 Å resolution Cp1 (this work) and the 1.0 Å resolution Av1 (PDB entry 3u7q) structures are used in structural comparison between the Cp1 and Av1 proteins. The mechanistic implications of the similarities and differences between the Cp1, Av1 and Kp1 proteins are also discussed.

## 2. Materials and methods   

### 2.1. Protein purification and crystallization   

Purification of Cp1 was performed as described previously (Kim *et al.*, 1993 ▶). Cp1 was crystallized using sitting-drop vapor diffusion with a reservoir solution consisting of 0.2 *M* lithium citrate, 20%(*w*/*v*) polyethylene glycol (PEG) 3350 at room temperature (∼295 K) in a Coy anaerobic chamber. The Cp1 crystals grown under this crystallization condition belonged to space group *P*2_1_, with unit-cell parameters *a* = 72.7, *b* = 170.6, *c* = 87.5 Å, β = 91.6°. The crystals were soaked in precipitation solution containing 10% 2-methyl-2,4-pentanediol (MPD) and 5 m*M* sodium dithionite for 15 min before flash-cooling them in liquid nitrogen.

### 2.2. Data collection and processing   

Crystallographic diffraction data were collected on beamline 12-2 at the Stanford Synchrotron Radiation Lightsource (SSRL) with a PILATUS 6M pixel-array detector. The 1.08 Å resolution diffraction data set was collected at 14 000 eV using an oscillation angle of 0.15°. Full sets of anomalous diffraction data for experimental phasing were collected at 7117 and 7130 eV, energies corresponding to the inflection point and above the Fe *K*-edge absorption edge, respectively, with an oscillation angle of 0.15° and using standard and inverse-beam modes of data collection. For site-specific X-ray absorption spectroscopy (Einsle *et al.*, 2007 ▶), multiwavelength anomalous diffraction (MAD) data were collected at different energies across the Fe *K*-edge absorption edge with an oscillation angle of 0.5° and an overall oscillation range of 360° at each energy point. A reference set of 360 diffraction images was collected at 12 658 eV with an oscillation angle of 0.5°.

The crystallographic data were processed using the *XDS* program package (Kabsch, 2010 ▶) and the *CCP*4 program package (Winn *et al.*, 2011 ▶). The phasing information was obtained from the MAD data (7117 and 7130 eV at the inflection and above the Fe *K*-edge absorption edge, respectively) using *SHELXC*/*D*/*E* (Sheldrick, 2008 ▶). The model was built manually with *Coot*, using the electron-density map from experimental phasing and using the protein sequence reported earlier as a guide (Kim *et al.*, 1993 ▶; Emsley *et al.*, 2010 ▶). The structure was further refined using *REFMAC*5 (Murshudov *et al.*, 2011 ▶). The models for the metalloclusters in Cp1 were built into the crystallographic structure based on the location of the peaks in the positive *F*_o_ − *F*_c_ difference density map and the anomalous density map. The models were then refined either without any restraints or using restraints generated from the 1.0 Å resolution Av1 structure (Spatzal *et al.*, 2011 ▶). These two refinement approaches resulted in essentially identical metalloclusters. The data-collection and refinement statistics are summarized in Tables 1 ▶ and 2 ▶. The electron-density analysis was carried out using in-house software. The structure validation analysis was performed using *MolProbity* v.4.02 (Chen *et al.*, 2010 ▶). The intermolecular contacts were analyzed using the *CCP*4 program *CONTACT* (Winn *et al.*, 2011 ▶). *PyMOL* was used to prepare the figures (DeLano, 2002 ▶).

The Fe *K*-edge site-specific X-ray absorption spectroscopy data were processed as described previously (Einsle *et al.*, 2007 ▶). Molybdenum and sulfur were also included in the anomalous refinement procedure owing to their significant anomalous signal in the Fe *K*-edge region; since the data sets were collected at energies remote from the S *K* absorption edge, a single set of Δ*f*′ and Δ*f*′′ parameters was used for all S atoms.

### 2.3. Coordinates   

Atomic coordinates and structure factors have been deposited in the Research Collaboratory for Structural Bioinformatics Protein Data Bank as entry 4wes.

## 3. Results and discussion   

### 3.1. Overall structure   

**Figure 1:**
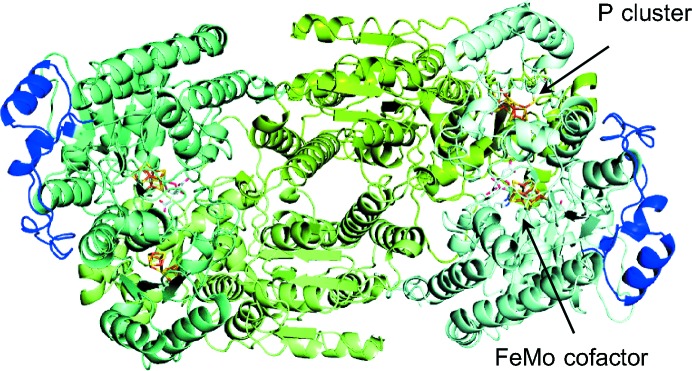
Ribbon representation of the Cp1 tetramer viewed down the molecular (noncrystallographic) twofold symmetry axis. The α-subunits are shaded in pale cyan and teal, except for the long insertion loops (residues α376–α429), which are highlighted in blue. The β-subunits are colored split pea and pale green. The FeMo cofactors and the P clusters are depicted as sticks, with C atoms shown in light gray, N atoms in blue, O atoms in red, S atoms in yellow, Fe atoms in orange and Mo atoms in cyan.

The 1.08 Å resolution X-ray crystal structure of Cp1 was determined by experimental phasing using MAD data (see §[Sec sec2]2 for details). The crystal form of Cp1 is similar to the previously reported cesium-derivative Cp1 structure in space group *P*2_1_, with unit-cell parameters *a* = 72.7, *b* = 170.6, *c* = 87.5 Å, β = 91.6° (Kim *et al.*, 1993 ▶). The data-processing and refinement statistics are summarized in Tables 1 ▶ and 2 ▶, respectively. An overview of the Cp1 tetramer structure is shown in Fig. 1 ▶. A total of 1953 residues (out of 1982 residues) and 2484 water molecules were included in the model for structural refinement. The first two residues in the N-terminus and the last 11–12 residues in the C-terminus of the α-subunit were not included in the model owing to weak electron density. The overall coordinate error in the model is estimated to be 0.022 Å from the diffraction-component precision index (Cruickshank, 1999 ▶). A single nonproline *cis*-peptide bond, first reported in Kp1 between Trpα251 and Serα252, is also present in Cp1 and Av1 (Leuα240–Thrα241 in Cp1 and Trpα253–Serα254 in Av1; Mayer *et al.*, 1999 ▶; Spatzal *et al.*, 2011 ▶). This pair of residues is located in a β-sheet of the second domain in the α-subunit (Kim & Rees, 1992 ▶), about 9 Å away from the FeMo cofactor. A few registry errors were corrected from the previously reported 3.0 Å resolution Cp1 structure, among which the longest (residues 412–420) is within the α-subunit insertion sequence characteristic of the group II MoFe proteins (Supplementary Fig. S1; Kim *et al.*, 1993 ▶).

With the exceptions of the long insertion (residues 376–429 in Cp1) and deletion (residues 1–47 in Av1) in the α-subunit and β-subunit, respectively, the overall structures of the Cp1 and Av1 folds are similar and can be superimposed with a root-mean-square deviation (r.m.s.d.) of the main chain of 0.92 Å for 1554 aligned residues (78% of the Cp1 sequence; Supplementary Fig. S2). For comparison, the overall r.m.s.d. deviation is 0.46 Å between the two closely related MoFe proteins Av1 and Kp1 over 1976 residues (99% of the Kp1 sequence). In addition to these ∼50-residue insertion/deletion regions, shorter gaps are present around residues 202–207 in the α-subunit and residues 162–168 in the β-subunit of Cp1 (both of which correspond to residues ∼210–220 in Av1).

### 3.2. Insertion sequence and the Fe protein docking site   

The long insert (residues 376–429) in the α-subunit of Cp1 adopts an irregularly structured loop involving residues 386–408 that is flanked by a short helix on each end (Supplementary Fig. S3). As it is positioned on the surface adjacent to the FeMo cofactor and close to the binding site on Av1 for the Fe protein, it has been speculated that this insertion may prevent Cp1 from forming an active complex with any orthologous Fe proteins (Kim *et al.*, 1993 ▶). Indeed, when superimposing the Cp1 and Cp2 (PDB entry 1cp2) structures onto different structures of the Av1–Av2 nitrogenase complex (PDB entries 1n2c, 2afk and 2afh; Tezcan *et al.*, 2005 ▶; Schindelin *et al.*, 1997 ▶; Schlessman *et al.*, 1998 ▶), the presumed Fe protein docking site is occluded by residues 386–392 in Cp1. This suggests that the interaction between Cp1 and Cp2 involves either a different Fe protein footprint or that the insertion loop must rearrange. The insertion sequence forms relatively few contacts with the rest of the protein, primarily mediated by residues in the α386–α408 region (Supplementary Table S1). These contacts can be grouped into three clusters, as shown in Supplementary Fig. S3. Additionally, intermolecular contacts are observed between residues in the insertion sequence and residues from adjacent molecules in the crystal lattice. This suggests that the insertion sequence may be relatively dynamic in solution, and thus may give the opportunity for Cp2 binding to Cp1 at the well conserved Fe protein docking site designated in the Av1–Av2 complex, while the conformation observed in the crystallographic structure may be stabilized as a result of crystal packing.

### 3.3. Structure of the metalloclusters   

#### 3.3.1. The FeMo cofactor and surrounding environment   

**Figure 2:**
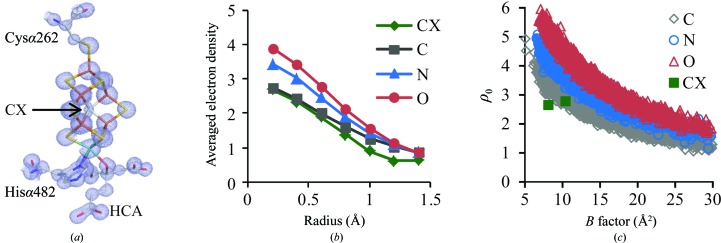
Characterization of the interstitial ligand in the Cp1 FeMo cofactor. (*a*) The refined crystallographic structure of the FeMo cofactor with the superimposed 2*F*_o_ − *F*_c_ electron-density map highlighted in light blue and contoured at 3σ. The interstitial ligand is modeled as carbon and labeled CX. C atoms are shown in light gray, N atoms in blue, O atoms in red, S atoms in yellow, Fe atoms in orange and Mo atoms in cyan. Homocitrate is labeled HCA. (*b*) The averaged electron density ρ(*r*) of the two crystallographically independent interstitial ligands CX (green) calculated within spheres of the indicated radii and compared with those calculated for proteinaceous C (gray), N (blue) and O (red) atoms with an isotropic *B* factor no greater than 30 Å^2^. (*c*) The variation in electron density (ρ_0_) at the atomic center as a function of the isotropic *B* factor for proteinaceous carbon (gray), nitrogen (blue) and oxygen (red). Atoms with isotropic *B *factors of <30 Å^2^ were included in the calculation. The data points representing two crystallographically independent interstitial ligands CX are shown in green.

A significant advantage of atomic resolution crystal structures is to minimize the influence of series-termination effects in Fourier maps, which are of particular concern for the interstitial ligand of the FeMo cofactor, as it is surrounded by six equidistant irons (Spatzal *et al.*, 2011 ▶; Einsle *et al.*, 2002 ▶). In the 2*F*_o_ − *F*_c_ map of the 1.08 Å resolution Cp1 structure, the electron density at the center of the FeMo cofactor clearly indicates the presence of an interstitial ligand in the cofactor of Cp1 (Fig. 2 ▶ *a*). Using the electron-density analyses developed previously (Spatzal *et al.*, 2011 ▶), the interstitial ligand has similar properties in both Cp1 and Av1, consistent with the assignment of this atom as carbon. Comparison of the averaged electron density ρ(*r*) calculated within spheres of different radii around a given atom type shows that the interstitial ligand in the FeMo cofactor most closely resembles proteinaceous carbon, but not nitrogen or oxygen (Fig. 2 ▶ *b*). The deviation of ρ(*r*) for the interstitial ligand from that of proteinaceous carbon at larger radius (>0.8 Å) may reflect the truncation error caused by the surrounding heavy atoms, such as Fe atoms, which are about 2 Å away from the interstitial ligand. In addition, the correlation between the electron density at the center of a given type of atom and the isotropic *B* factor of the interstitial ligand also falls in the range of proteinaceous carbon, but not nitrogen or oxygen (Fig. 2 ▶ *c*).

**Figure 3:**
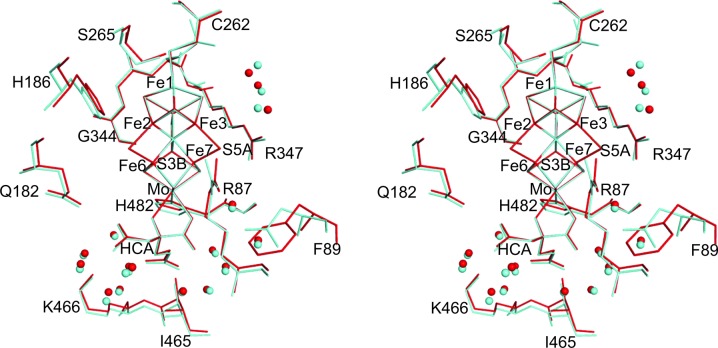
Stereoview of a stick representation of the FeMo cofactor environment in Cp1 (red) and Av1 (cyan). For clarity, only the residues and water molecules participating in polar interactions with the cofactor are shown. The amino-acid residues are represented by thin lines and waters are shown as spheres.

As summarized in Table 3 ▶ (and detailed in Supplementary Table S2), the metal–ligand and metal⋯metal distances of the FeMo cofactor in Cp1 and Av1 are essentially the same. The largest difference is observed between the C1 carboxyl groups of the homocitrate (Fig. 3 ▶), which leads to O2 of the homocitrate in Cp1 being about 0.3 Å closer to Fe6 of the FeMo cofactor. Interestingly, a more significant displacement (0.7 Å away from Fe6) in the C1 carboxyl group of the homocitrate has been reported recently in Av1 at high pH, and the C1 arm has been proposed to possibly play a role in proton transfer (Howard & Rees, 1994 ▶; Yang *et al.*, 2014 ▶).

Residues making side-chain contacts to the FeMo cofactor are highly conserved in all groups of nitrogenases (Howard *et al.*, 2013 ▶). Among these residues, those potentially forming hydrogen bonds or other polar contacts with the cofactor through either the peptide backbone or side chains are highlighted in Fig. 3 ▶. Of the five residues with side chains mediating polar interactions, four are invariant in both the group I and group II MoFe proteins (Howard *et al.*, 2013 ▶). The fifth residue, Lysα466 (Lysα426 in Av1), is strictly conserved in group I and is a dominant single variant with an Arg found in two cases in group II. The strict conservation of these residues emphasizes their important roles for the function of nitrogenase.

A subtle, but intriguing, difference between Cp1 and Av1 is observed for residue Argα87 (Argα96 in Av1) in proximity to the FeMo cofactor. Arg*α*87 is conserved with a single variant (Lys) in all groups of MoFe proteins. In both high-resolution Av1 structures this Arg consistently shows a significant non­planar distortion of the guanidinium group, characterized by an averaged CD—NE—CZ—NH2 torsion angle of around 26° (Spatzal *et al.*, 2011 ▶; Einsle *et al.*, 2002 ▶). The equivalent torsion angle in Cp1 is ∼6°, indicating a more planar group. As a consequence, there are slight differences in hydrogen-bonding geometry between the side chain of this residue and S5A of the cofactor. In addition, in Av1 but not in Cp1 this arginine residue also hydrogen bonds to the side chain of Asn*α*98; the equivalent residue in Cp1 (Pheα89) would not support a hydrogen-bond interaction.

Four water molecules adjacent to the FeMo cofactor are conserved in Cp1, Av1 and Kp1, aligned along the Fe3–Fe7 side and within 4.0 Å of cluster sulfurs (Fig. 3 ▶; Spatzal *et al.*, 2013 ▶; Mayer *et al.*, 1999 ▶). In addition, there is also a large water pool (∼30 water molecules) around the homocitrate of the FeMo cofactor conserved in Cp1, Av1 and Kp1, 13 of which directly interact with the homocitrate (Fig. 3 ▶). This pool extends between the FeMo cofactor and the P cluster (see discussion later in this paper).

#### 3.3.2. The P cluster and surrounding environment   

**Figure 4:**
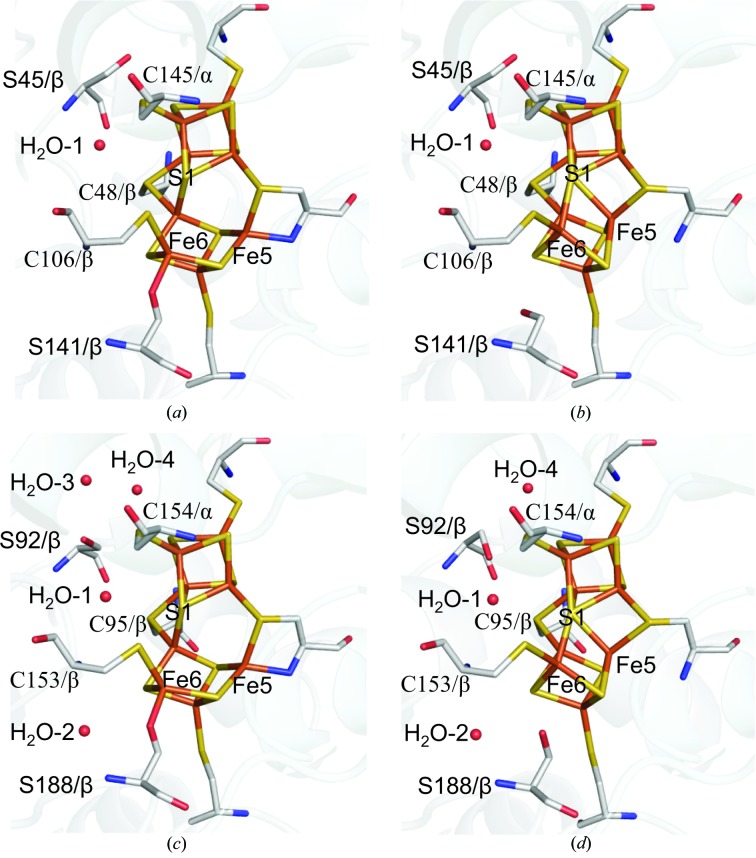
Conformational states of the P cluster in Cp1 and Av1. The P clusters in the Cp1 structure reflect a superposition of the two conformations corresponding to P^OX^ (*a*) and P^N^ (*b*) present at a ratio of 6:4, respectively. The P clusters of the Av1 P^OX^ (1.0 Å resolution; PDB entry 3u7q) and P^N^ (1.16 Å resolution; PDB entry 1m1n) conformations are shown in (*c*) and (*d*), respectively. The backbone atoms of the α-subunits and β-subunits are represented as ribbons shaded light blue and gray, respectively. The P cluster and coordinated ligand residues as well as Serβ45 in Cp1 (Serβ188 in Av1) are represented as sticks. The waters are shown as spheres. C atoms are highlighted in gray, N atoms in blue, O atoms in red, S atoms in yellow and Fe atoms in orange.

The Av1 and Kp1 structural studies have established that the P cluster can adopt at least two distinct conformational states that have been assigned as the resting (P^N^) and two-electron oxidized (P^OX^) states (Peters *et al.*, 1997 ▶; Mayer *et al.*, 1999 ▶). While these conformations will be described as P^N^ and P^OX^, it is worth noting that these assignments should be considered tentative and have not been conclusively corroborated by spectroscopy of the crystalline samples. In the P^N^ state, all Fe atoms in the P cluster are coordinated exclusively by sulfur. Of note, S1 bridging the two 4Fe–3S partial cubanes is coordinated to six Fe atoms, while the P cluster is coordinated to the protein through the side-chain sulfurs of six invariant cysteines from the α-subunits and β-subunits. Upon oxidation, Fe5 and Fe6 move away from S1, and the coordinating interactions are replaced by the amide group of Cysα88 and the hydroxyl group of Serβ188 in Av1 (corresponding to Cysα79 and Serβ141 in Cp1), respectively (Peters *et al.*, 1997 ▶; Mayer *et al.*, 1999 ▶). Electron-density analysis and model fitting indicate that the P cluster in Cp1 corresponds to a mixture of the P^N^ and P^OX^ conformations, as was also observed in the 1.0 Å resolution Av1 structure (Spatzal *et al.*, 2011 ▶; Peters *et al.*, 1997 ▶; Mayer *et al.*, 1999 ▶). As shown in Supplementary Fig. S4, a pronounced peanut-shaped density is observed around Fe5 and Fe6 in the 2*F*_o_ − *F*_c_ map, while the density for all other Fe atoms in the P cluster displays a well defined spherical shape. This can be modeled as a mixture of the P^N^ and P^OX^ states in a ratio of 4:6 (Mayer *et al.*, 1999 ▶; Peters *et al.*, 1997 ▶), with Fe5 and Fe6 moving ∼1.1 Å away from S1 upon oxidation. In the 1.0 Å resolution Av1 structure, the oxidized state is more populated (∼2:8 P^N^:P^OX^) and Fe5/Fe6 appear marginally further (∼0.1 Å) away from S1 than in Cp1 (Supplementary Table S2; Spatzal *et al.*, 2011 ▶). The remainder of the P-cluster structure in Av1 and Cp1 are quite similar, with an overall r.m.s.d. of 0.05 Å when Fe5 and Fe6 are excluded. The side chain of Serβ141 in Cp1 also shows two alternative conformations with the hydroxyl-group O atoms positioned 2.1 Å apart, correlated with the alternative conformations of Fe6 in the P cluster (Fig. 4 ▶). Alternative conformations for this residue are not observed in either Av1 or Kp1. Instead, there is a water molecule (H_2_O-2) at around 3.3 Å from the hydroxyl O atom of Serβ188 in Av1 and Kp1, but not in Cp1 (Fig. 4 ▶).

The non-S protein ligands in the P^OX^ structure, Cysα79 NH and Serβ141 OH, are ionizable and could potentially contribute to proton-coupled electron transfer and pH-dependence of the P cluster reduction potential (Lanzilotta *et al.*, 1998 ▶). Remarkably, both residues can be substituted without loss of function in Av1, in contrast to the other cysteine residues coordinating the remaining Fe in the P cluster (Dean *et al.*, 1990 ▶). In P^OX^, Fe5 is not coplanar with the coordinating amide group of Cysα79 in any of the three high-resolution MoFe protein structures, which has been interpreted as an indicator of a protonated amide N serving as the Fe5 ligand (Mayer *et al.*, 1999 ▶; Spatzal *et al.*, 2011 ▶). However, a small-molecule compound with a similarly distorted tetrahedral Fe site containing a non-coplanar, deprotonated pyrazole nitrogen ligand has been characterized (Milione *et al.*, 2009 ▶). An additional consideration is that a protonated carboxamido N would likely be *sp* ^3^-hybridized to also serve as a metal ligand, thereby resulting in distortion of the Glyα87–Cysα88 peptide bond, which is not observed in Cp1, Av1 or Kp1. As the upper limit of the p*K*_a_ of a metal-bound carboxamide is typically around 4.5 (Noveron *et al.*, 2001 ▶; Tyler *et al.*, 2003 ▶), it is most likely that a deprotonated amide N atom coordinates Fe5 in P^OX^ in a distorted, non-coplanar fashion. The side chain of Serβ141 coordinated to Fe6 in P^OX^ (corresponding to Serβ188 in Av1) is also likely to be deprotonated. This is supported by the absence of any tetrahedral Fe complex in the Cambridge Crystallographic Structure Database (CCDC) coordinating a protonated organic hydroxyl group (Allen, 2002 ▶). Furthermore, the Fe6—O distances in Cp1 and Av1 (1.93 and 1.91 Å, respectively; Supplementary Table S3) are well within the range of the Fe—O bond lengths in tetrahedral Fe compounds with deprotonated organic hydroxyl-group ligands (1.90 ± 0.07 Å for 105 hits with *R* factor ≤ 0.075).

Serβ45 (corresponding to Serβ92 in Av1 and Serβ90 in Kp1) is another residue near the P cluster that may undergo oxidation-state-induced conformational changes. This residue is invariant in the group I and group II MoFe proteins, while either a double variant (Ser/Ala/Gly) or an invariant Gly has been found in other groups (Howard *et al.*, 2013 ▶). Two different rotamers for the side chain of this Ser residue have been observed in Cp1 and Av1 (Fig. 4 ▶). In the present Cp1 and the 1.0 Å resolution Av1 structure, the hydroxyl O of this residue faces away from the P cluster (χ_1_ angle of 169.5° in Cp1 and 121.4° in Av1) and connects to the surface water pool. In the 1.16 Å resolution Av1 structure, where the P cluster is in the P^N^ state, a different rotamer (χ_1_ angle of 40.7°) is observed, permitting potential hydrogen-bond formation to S2A and a conserved water molecule (H_2_O-1). Considering the location and the conformational alternations observed at this residue, it is reasonable to propose that Serβ45 may serve as a proton shuttle from the water pool at the protein surface to the P cluster (Fig. 4 ▶).

#### 3.3.3. Hydrogen-bonding network between the P cluster and the FeMo cofactor   

The tertiary structural alignment of the MoFe proteins identifies a conserved water tunnel between the water pool around the homocitrate of the FeMo cofactor and the P cluster Fe3 in Cp1, Av1 and Kp1, and it may potentially connect the two metalloclusters through a hydrogen-bonding network (Spatzal *et al.*, 2011 ▶; Mayer *et al.*, 1999 ▶). This water tunnel is surrounded by two short and two long helices: the short helices, involving residues α54–α64 and residues α77–α83 in Cp1, are approximately parallel to the tunnel, while the two long helices, corresponding to residues α182–α196 and residues β46–β60 in Cp1, are roughly perpendicular to the tunnel (Supplementary Fig. S6; Kim *et al.*, 1993 ▶). Such conserved structural arrangements may facilitate electron/proton-transfer process between the two metallo­clusters (Gray & Winkler, 2005 ▶; Markovitch *et al.*, 2008 ▶).

### 3.4. The Fe16 site   

**Figure 5:**
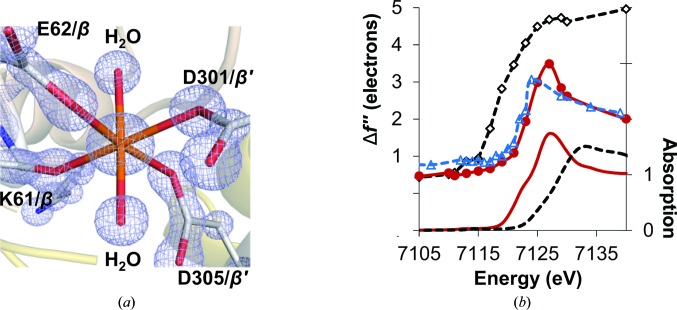
Characterization of the MMB site. (*a*) The 2*F*_o_ − *F*_c_ electron-density map (light blue) at the MMB site, with Fe16 and coordinating ligands contoured at 3σ. The C atoms are highlighted in gray, N atoms in blue, O atoms in red and Fe atoms in orange. (*b*) Top: comparison of the refined Δ*f*′′ spectrum of Fe16 (red solid line with red circles) with that of the averaged Fe in the P cluster (black dashed line with white diamonds) of Cp1 and Fe16 in Av1 (blue dashed line with blue triangles). Bottom: the XAS spectra of ferrous sulfate heptahydrate (FeSO_4_.7H_2_O, red solid line) and ferric sulfate hydrate [Fe_2_(SO_4_)_3_.*x*H_2_O, black broken line] (Zhang *et al.*, 2013 ▶). No background removal or normalization were applied to the refined Δ*f*′′ spectra.

Recently, we have reported a 16th Fe (designated Fe16) in the mononuclear metal-binding site (MMB site) of Av1 between the two β-subunits (Zhang *et al.*, 2013 ▶). Fe16 in Av1, a partially occupied ferrous Fe, exhibits an approximately octa­hedral geometry coordinated by the side-chain O atoms of three Glu/Asp residues, the backbone carbonyl O atom of Arg and two water molecules. The variation of the three coordinating side-chain residues among all six groups of MoFe proteins is minor and the variants are also capable of providing oxygen ligands (Howard *et al.*, 2013 ▶). The metal at the MMB site of Cp1 adopts a similar coordination environment as in Av1, with a Lys instead of an Arg providing the carbonyl oxygen ligand (Fig. 5 ▶ *a*). The site-specific Fe *K*-edge X-ray absorption near-edge spectrum of Fe16 in Cp1 matches those of both Av1 and ferrous sulfate well, but not that of ferric sulfate, indicating that Fe16 in Cp1 is also a ferrous Fe (Fig. 5 ▶ *b*; Zhang *et al.*, 2013 ▶). The occupancy of Fe16 in Cp1 was quantified by electron density and by an Fe *K* absorption-edge jump as described previously using seven sets of MAD data collected from different Cp1 crystals (Zhang *et al.*, 2013 ▶). We found, using either method, that Fe16 in Cp1 is about half occupied with little variation between crystals (Supplementary Table S4). This is a significantly different situation from that we observed in Av1, where the occupancy of Fe16 essentially varied from 0 to 1 among the eight sets of MAD data examined. Another difference in the Fe16 sites between Av1 and Cp1 is that the alternative conformations observed in the Av1 MMB site are not evident in Cp1 (Fig. 5 ▶ *a*; Zhang *et al.*, 2013 ▶). This difference could be explained by the presence of Li^+^ in the crystallization solution of Cp1. The average metal–O distance for Fe16 (2.09 Å) in Cp1 is similar to the average Li–O distances of Li^+^ compounds (2.15 ± 0.08 Å for 197 LiO_6_ compounds with *R* factor ≤ 0.075) found in the CCDC (Allen, 2002 ▶); therefore, a Li or Fe ion may share similar coordination environment.

Fe16 is approximately 24 and 21 Å away from the P cluster and the FeMo cofactor, respectively, and may be structurally coupled to the two metalloclusters through two short α-helices. Lysβ61 (Argβ107 in Av1) binds to Fe16 though the carbonyl O atom and is located at the C-terminal end of a β-subunit helix involving residues β45–β61 in Cp1 (corresponding to residues β92–β108 in Av1), extending between the FeMo cofactor and the P cluster. Residue Argβ58 (Argβ105 in Av1) in this helix interacts with O6 of the homocitrate *via* a conserved water molecule (Supplementary Fig. S5). At the N-terminal end of this helix, Cysβ48 binds to Fe2 and Fe8 in the P cluster. An alternative connection involves an α-subunit helix (residues α465–α473 in Cp1 and residues α425–α433 in Av1). Lysα473 (Lysα433 in Av1) is about 4.4 Å away from the Fe16 site, while at the other end of this helix, Lysα466 (Lysα426 in Av1) and Ileα465 (Ileα425 in Av1) hydrogen-bond to the C6 carboxyl group of homocitrate through the side chain or backbone amide group, respectively. The residues mentioned above are either invariant (Lysα473 and Cysβ48) or single variants (Lysα466 and Argβ58) with a similar type of amino acid among all six groups of nitrogenases (Howard *et al.*, 2013 ▶). In addition, the three-dimensional structural arrangements connecting Fe16 and the two metalloclusters are well conserved in Cp1, Av1 and Kp1.

## 4. Conclusions   

In this study, we have characterized the X-ray crystal structure of Cp1 at atomic resolution and carried out a detailed comparison with the Av1 structure determined at 1.0 Å resolution. Cp1 and Av1 are representatives of the two distinct groups of MoFe proteins (∼37% sequence identity), differing primarily by a long insertion in the α-subunit and a deletion in the β-subunit of Cp1 relative to Av1. Our analysis confirms that the metal centers, the FeMo cofactor and the P cluster metallocluster, and the Fe16 mononuclear metal-binding site are essentially identical in Av1 and Cp1. The structural arrangements immediately outside the coordination shell of the FeMo cofactor are highly conserved between the two MoFe proteins, indicating that such motifs are critical for the function of the enzyme. More pronounced differences are observed around the P cluster of the two proteins, suggesting that the mechanism of nitrogen fixation is less sensitive to perturbations in this region. Av1 and Cp1 share a conserved water tunnel and similar secondary-structure elements between the P cluster and the FeMo cofactor, indicating that such a structural arrangement is crucial for the interactions between these two clusters. The most significant differences are evident in the Fe protein docking surface of the MoFe protein, which is occluded in Cp1, relative to Av1 and Kp1, by the long α-subunit sequence insertion. As this loop forms few contacts with the remainder of the protein, it is plausible that it rearranges during complex formation to permit similar interactions during turnover between the MoFe protein and the Fe protein of different organisms.

Nitrogenase is the only validated biological system capable of fixing nitrogen; the complexity of the biosynthetic pathway and the difficulties in preparing synthetic homogeneous catalysts reinforce the conclusion that dinitrogen reduction under physiological conditions is a demanding reaction to achieve. Giving the challenges in developing a successful catalyst like nitrogenase, it is likely there are significant restrictions in the active-site variation that can be tolerated. A comparison of Cp1 with Av1 supports this view; although the overall sequence identity is ∼37%, the metal centers are essentially superimposable in these ∼1 Å resolution structures and the protein environment surrounding the active-site FeMo cofactor is highly conserved both in terms of sequence and structure. While the basic framework is maintained, greater variation is tolerated surrounding the P cluster and at the Fe protein docking surface, suggesting that intermolecular and intramolecular electron and proton transfers to the active site may be less sensitive to variations in sequence and structure.

## Supplementary Material

PDB reference: MoFe protein, 4wes

Supporting Information.. DOI: 10.1107/S1399004714025243/lp5003sup1.pdf

## Figures and Tables

**Table 1:** Summary of the data-processing statistics Values in parentheses are for the outer shell.

	14000eV† (0.886)	7130eV† (1.734)	7117eV† (1.742)
Space group	*P*2_1_		
Unit-cell parameters (, )	*a* = 72.70, *b* = 170.58, *c* = 87.54, = 91.63		
Resolution ()	38.91.08 (1.141.08)	42.672.04 (2.172.04)	42.672.04 (2.172.04)
*R*_merge_ (%)	6.4 (80.3)	2.9 (5.4)	2.7 (4.9)
*R*_p.i.m._ (%)	3.9 (49.4)	1.7 (3.4)	1.3 (2.3)
Unique reflections	842587 (117365)	124980 (15125)	120979 (14139)
Multiplicity	3.6 (3.6)	6.8 (6.4)	6.8 (6.4)
Completeness (%)	93.0 (88.7)	92.5 (76.8)	91.4 (73.3)
*I*/**(*I*)	9.7 (1.6)	42.8 (22.9)	46.8 (25.6)
Anomalous completeness (%)		90.1 (73.2)	91.4 (73.3)
Anomalous multiplicity		3.5 (3.4)	3.5 (3.4)

† The X-ray energies (wavelengths) at which the diffraction data were collected.

**Table 2:** Summary of refinement statistics

Resolution ()	38.91.08
*R*_work_ (%)	11.0
*R*_free_ (%)	13.3
R.m.s.d., bonds ()	0.012
R.m.s.d., angles ()	1.64
Average *B* factor (^2^)	
Protein and ligands	15.3
Solvent	33.7
Diffraction precision index ()	0.022
Ramachandran statistics (%)	
Favored	97.3
Allowed	2.55
Outliers	0.15
Missing residues (residue Nos.)	
-Subunit	1, 2, 522533
-Subunit	1, 2, 521533

**Table 3:** Comparison of the metalligand and metalmetal distances in the FeMo cofactors of Cp1 and Av1

	Cp1	Av1
FeS	2.25 0.03	2.24 0.03
FeC	2.00 0.02	2.00 0.01
MoS	2.36 0.01	2.36 0.01
MoN	2.35 0.01	2.29 0.02
MoO	2.20 0.03	2.19 0.02
Short FeFe†	2.64 0.04	2.63 0.03
Long FeFe‡	3.69 0.01	3.70 0.01
Fe1FeFe	4.97 0.01	5.00 0.01
FeMo	2.68 0.01	2.69 0.03
FeFeMo	5.04 0.01	5.06 0.02
Fe1FeFeMo	6.95 0.00	7.00 0.00

† The distances between Fe within each half of the FeMo cofactor (*i.e.* FeFe distances involving Fe1Fe4 and FeFe distances involving Fe5Fe7) and between the belt Fe atoms located in the two distinct halves of the FeMo cofactor, with the FeFe vector parallel to the Fe1Mo axis (*i.e.* Fe2 and Fe6, Fe3 and Fe7, and Fe4 and Fe5).

‡ The distances between the belt Fe atoms located in the two different halves of the FeMo cofactor, with the FeFe vector not parallel to the Fe1Mo axis (*i.e.* between Fe2 and Fe5/7, between Fe3 and Fe5/6, and between Fe4 and Fe6/7).
