# Supplementary material for: Nitrogenase MoFe protein from Clostridium pasteurianum at 1.08 Å resolution: comparison with the Azotobacter vinelandii MoFe protein
Source: Acta Crystallogr D Biol Crystallogr. 2015 Jan 23;71(Pt 2):274–82. doi: 10.1107/S1399004714025243 (PMC4321486; doi:10.1107/S1399004714025243)
Supplement: Supplementary file 1 [file d-71-00274-sup1.pdf]

Acta Cryst D

BIOLOGICAL CRYSTALLOGRAPHY

Volume 71 (2015)

Supporting information for article:

Nitrogenase MoFe protein from *Clostridium pasteurianum* at 1.08 Å resolution: comparison with the *Azotobacter vinelandii* MoFe protein

Li-Mei Zhang, Christine N. Morrison, Jens T. Kaiser and Douglas C. Rees

Supplementary Materials for

Nitrogenase MoFe-Protein from *Clostridium pasteurianum* at 1.08 Å Resolution: Comparison to the *Azotobacter vinelandii* MoFe-protein

Li-Mei Zhang[a], Christine N. Morrison[a], Jens T. Kaiser[a], Douglas C. Rees[a,b*]

[a]Division of Chemistry and Chemical Engineering, and [b]Howard Hughes Medical Institute, California Institute of Technology, Pasadena CA 91125, USA.

[*]Correspondence to Douglas C. Rees: dcrees@caltech.edu, Division of Chemistry and Chemical Engineering, and Howard Hughes Medical Institute, California Institute of Technology, MC 164-96, 1200 E. California Blvd, Pasadena CA 91125, USA.

Supplementary Tables

Table S1. Polar interactions between residues α386 – α408 in the irregularly structured insertion loop of the α-subunit and the rest of the Cp1 structure. A distance cutoff of 4.0 Å was used for this calculation.

**Insertion Loop Residues**		**α-Subunit Residues**		**Distance (Å)**
Asp	α389-OD2	Arg	α42-NE	2.93
Pro	α394-O	Asn	α191-OD1	2.95
Pro	α394-O	His	α187-NE2	3.28
Asp	α401-N	Lys	α276-O	2.9
Lys	α404-NZ	Gly	α199-O	2.98
Arg	α406-O	Tyr	α277-O	3.47
Arg	α406-N	Tyr	α277-O	3.04
Arg	α406-NH1	Gln	α205-OE1	3.37
Arg	α406-NH1	Asp	α253-O	2.8
Arg	α406-NH2	Tyr	α277-O	3.45
**Insertion Loop Residues**		**β-Subunit Residues**		**Distance (Å)**
Asp	α389-OD1	Thr	β72-OG1	3.95
Asn	α392-ND2	Glu	β73-OE2	3.65

Table S2. Comparison of interatomic distances in the FeMo-cofactors of Cp1 and Av1.

	Bond Distances[a] (Å)			Metal···metal Distances[a] (Å)	
	**Cp1**	**Av1**		**Cp1**	**Av1**
FE1—S1A	2.31	2.30	FE1···FE2	2.66	2.67
FE1—S2A	2.26	2.27	FE1···FE3	2.67	2.67
FE1—S4A	2.31	2.29	FE1···FE4	2.61	2.66
FE1—SG-Cys α272	2.28	2.27	FE2···FE3	2.68	2.67
FE2—CX	2.01	2.00	FE2···FE4	2.66	2.65
FE2—S1A	2.27	2.26	FE2···FE6	2.58	2.58
FE2—S2A	2.27	2.25	FE3···FE4	2.66	2.64
FE2—S2B	2.22	2.20	FE3···FE7	2.59	2.59
FE3—CX	2.01	1.99	FE4···FE5	2.58	2.61
FE3—S2A	2.29	2.27	FE5···FE6	2.63	2.63
FE3—S4A	2.28	2.25	FE5···FE7	2.63	2.63
FE3—S5A	2.23	2.23	FE6···FE7	2.59	2.60
FE4—CX	1.96*	2.00	Mo1···FE5	2.69	2.73
FE4—S1A	2.30	2.29	Mo1···FE6	2.68	2.67
FE4—S3A	2.23	2.24	Mo1···FE7	2.67	2.68
FE4—S4A	2.31	2.29			
FE5—CX	1.98	2.01	FE2···FE5	3.68	3.69
FE5—S1B	2.26	2.27	FE2···FE7	3.69	3.69
FE5—S3A	2.26	2.26	FE3···FE5	3.71	3.71
FE5—S4B	2.24	2.26	FE3···FE6	3.69	3.68
FE6—CX	2.02	2.00	FE4···FE6	3.70	3.72
FE6—S1B	2.23	2.24	FE4···FE7	3.69	3.69
FE6—S2B	2.18	2.17			
FE6—S3B	2.23	2.22	FE1···FE5	4.96	5.02
FE7—CX	2.00*	1.99	FE1···FE6	4.98	5.00
FE7—S3B	2.25	2.25	FE1···FE7	4.99	5.00
FE7—S4B	2.23	2.22	Mo1···FE2	5.03	5.03
FE7—S5A	2.20	2.21	Mo1···FE3	5.05	5.05
Mo1—ND1-His α482	2.34*	2.32	Mo1···FE4	5.04	5.09
Mo1—O5-HCA	2.22	2.20			
Mo1—O7-HCA	2.18*	2.17			
Mo1—S1B	2.35	2.36	Mo1···FE1	6.95	7.00
Mo1—S3B	2.37	2.37			
Mo1—S4B	2.35	2.35			

[a]The reported distances are the average between the non-crystallographically related pairs of the FeMo-cofactors in the Av1 and Cp1 structures, respectively. The standard deviations of the averaged distances are all smaller than 0.03 Å unless marked with * for those of 0.03 Å.

Table S3. Comparison of the interatomic distances in the P-clusters of Cp1 and Av1.

Bond Distances[a] (Å)			Metal···metal Distances (Å)		
	**Cp1**	**Av1**		**Cp1**	**Av1**
		Conformation A: P^{OX} form			
FE1—S1	2.47	2.48	FE1···FE2	2.50	2.53
FE1—S2A	2.30	2.31	FE1···FE3	2.76	2.75
FE1—S3A	2.30	2.29	FE1···FE4	2.63	2.57
FE1—SG-Cys β48	2.36	2.33	FE1···FE5	4.77	4.86
FE2—S1	2.39	2.38	FE1···FE6	5.63	5.85
FE2—S2A	2.33	2.32	FE1···FE7	5.34	5.48
FE2—S4A	2.33	2.32	FE1···FE8	2.87	2.94
FE2—SG-Cys α145	2.32	2.32	FE2···FE3	2.78	2.80
FE3—S2A	2.32	2.30	FE2···FE4	2.62	2.63
FE3—S3A	2.28	2.27	FE2···FE5	5.63	5.78
FE3—S4A	2.33	2.33	FE2···FE6	5.95	6.14
FE3—SG-Cys α53	2.30	2.29	FE2···FE7	6.67	6.80
FE4—S1	2.39	2.36	FE2···FE8	4.34	4.37
FE4—S3A	2.28	2.29	FE3···FE4	2.71	2.72
FE4—S4A	2.32	2.31	FE3···FE5	6.09	6.19
FE4—SG-Cys α79	2.34	2.31	FE3···FE6	7.76	7.98
FE5—N-Cys α79	2.12	2.11	FE3···FE7	7.56	7.71
FE5—S2B	2.19	2.28	FE3···FE8	5.42	5.48
FE5—S4B	2.37	2.31	FE4···FE5	3.60	3.69
FE5—SG-Cys α79	2.37	2.32	FE4···FE6	5.49	5.68
FE6—OG-Ser β141	1.93*	1.91	FE4···FE7	5.45	5.54
FE6—S2B	2.29	2.34	FE4···FE8	3.85	3.82
FE6—S3B	2.33	2.33	FE5···FE6	3.76	3.98
FE6—SG-Cys β106	2.41	2.39	FE5···FE7	2.77	2.83
FE7—S2B	2.30	2.32	FE5···FE8	3.39	3.43
FE7—S3B	2.32	2.33	FE6···FE7	2.76	2.80
FE7—S4B	2.32	2.29	FE6···FE8	3.21	3.30
FE7—SG-Cys β23	2.33	2.37	FE7···FE8	2.69	2.75
FE8—S1	2.36	2.36			
FE8—S3B	2.27	2.28			
FE8—S4B	2.32	2.32			
FE8—SG-Cys β48	2.31	2.32			
FE5···S1[a]	3.67	3.82			
FE6···S1[a]	3.65*	3.86			

Conformation B: P^N form					
FE5—S1	2.58	2.65*	FE5···FE1	3.83	3.91
FE5—S2B	2.45	2.43	FE5···FE2	4.63	4.72*
FE5—S4B	2.23	2.23	FE5···FE3	5.36	5.48
FE5—SG-Cys α79	2.28	2.27	FE5···FE4	2.89	2.96
FE6—S1	2.53	2.63	FE5···FE6	2.52	2.55*
FE6—S2B	2.50	2.52	FE5···FE7	2.75	2.79
FE6—S3B	2.36	2.31	FE5···FE8	2.51	2.49*
FE6—SG-Cys β106	2.27	2.26	FE6···FE1	4.55	4.65
			FE6···FE2	4.86	4.94
			FE6···FE3	6.64	6.75
			FE6···FE4	4.43	4.51
			FE6···FE7	2.78	2.81
			FE6···FE8	2.37	2.34

[a] The reported distances are the average between the non-crystallographically related pairs of the P-clusters in the Av1 and Cp1 structures, respectively. The standard deviations of the averaged distances are all smaller than 0.03 Å unless marked with * for those ranging from 0.03 to 0.05 Å.
[b]The distances between Fe5/6 and S1 in the P^{ox} conformation are included to show the changes of the distances relative to the P^N conformation.

Table S4. Estimation of the occupancy number of Fe16 in different Cp1 crystals by analysis of the MAD data.

Sample ID		1	2	3	4	5	6	7
**Protein Source**		PrepA	PrepB	PrepB	PrepB	PrepC	PrepC	PrepC
**Resolution Range[a] (Å)**		40-1.42	40 - 1.40	40-1.12	40 - 1.30	40 - 1.45	40 - 1.55	40 - 1.65
**B-Overall[b] ($Å^2$)**		19.08	19.06	16.29	17.94	18.34	18.24	18.72
**Edge Jump[c]**	**AvgClust Fe[d]**	4.79 (0.33)	4.77 (0.33)	4.36 (0.34)	4.49 (0.31)	4.33 (0.23)	4.32 (0.22)	4.61 (0.31)
	**Fe16**	2.37	2.49	2.18	2.44	2.07	2.22	2.15
	**Fe16/ AvgClust Fe**	0.49	0.52	0.50	0.54	0.48	0.51	0.47
**ON of Fe16[e]**		0.35	0.37	0.36	0.37	0.35	0.37	0.33
**Electron Density[f]**	**AvgClust Fe[d]**	5.64 (0.19)	5.65 (0.21)	6.60 (0.20)	5.45 (0.17)	5.69 (0.21)	5.79 (0.19)	5.85 (0.19)
	**Fe16**	3.05	3.17	3.5	3.03	2.98	3.02	3.18
	**Fe16/ AvgClust Fe**	0.55	0.56	0.53	0.56	0.52	0.52	0.54

[a]The resolution range is for the higher resolution reference diffraction data. The highest resolution limits for the anomalous diffraction data collected across Fe K-edge are 1.9 – 2.1 Å.
[b]The overall B-factor is calculated for all the atoms in the crystallographic structure.
[c]The edge jump is defined by the difference of $\Delta f''$ above and below Fe K-edge (at 7130 eV and 7080 eV respectively, except for Crystal #5-7 for which 7100 eV is used). All the Fe in the P-cluster and the FeMo-cofactor of Cp1 are used in calculating the averaged edge jump (AveClustFe).
[d]The numbers in parentheses are the standard deviations of the values listed in the same cell.
[e]The occupancy number (ON) of Fe16 is estimated by comparing the ratio of the edge jump of Fe16 to the average of the Fe in the metalloclusters with that of the normalized X-ray absorption spectra of ferrous sulfate heptahydrate to the MoFe-protein. When normalized at 7150 eV, the ratio of the defined edge jump between ferrous sulfate heptahydrate and the MoFe-protein is around 1.4.
[f]Fe5 and Fe6 in the P-cluster are excluded from the average due to the existence of alternative structures with variable occupancy at these two Fe sites.

Supplementary Figuures

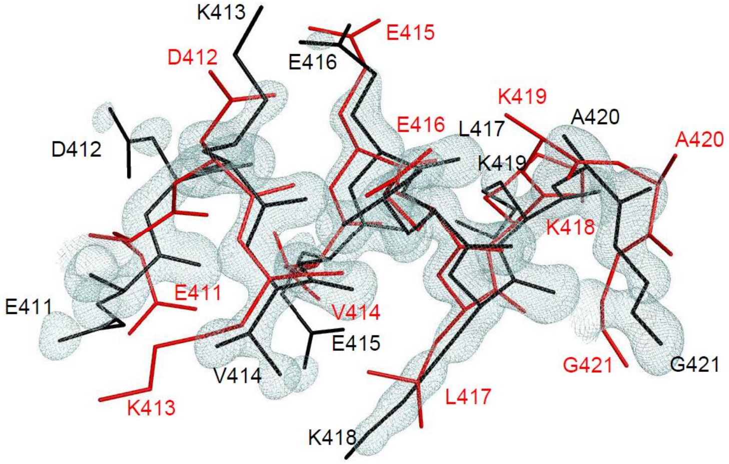

Figure S1. Registry correction to the previously reported Cp1 structure (PDB ID 1MIO) in the long insertion loop of the α-subunit. The Cp1 structure refined at 1.08 Å resolution in the present work is depicted with black bonds, and the 1MIO structure is highlighted in red. The electron density map of the 1.08 Å resolution Cp1 structure is displayed at a contour level of 1.5 σ in paleblue color. The registry error was initiated by mis-placing D412 into K413 residue position, and extends to residue A420.

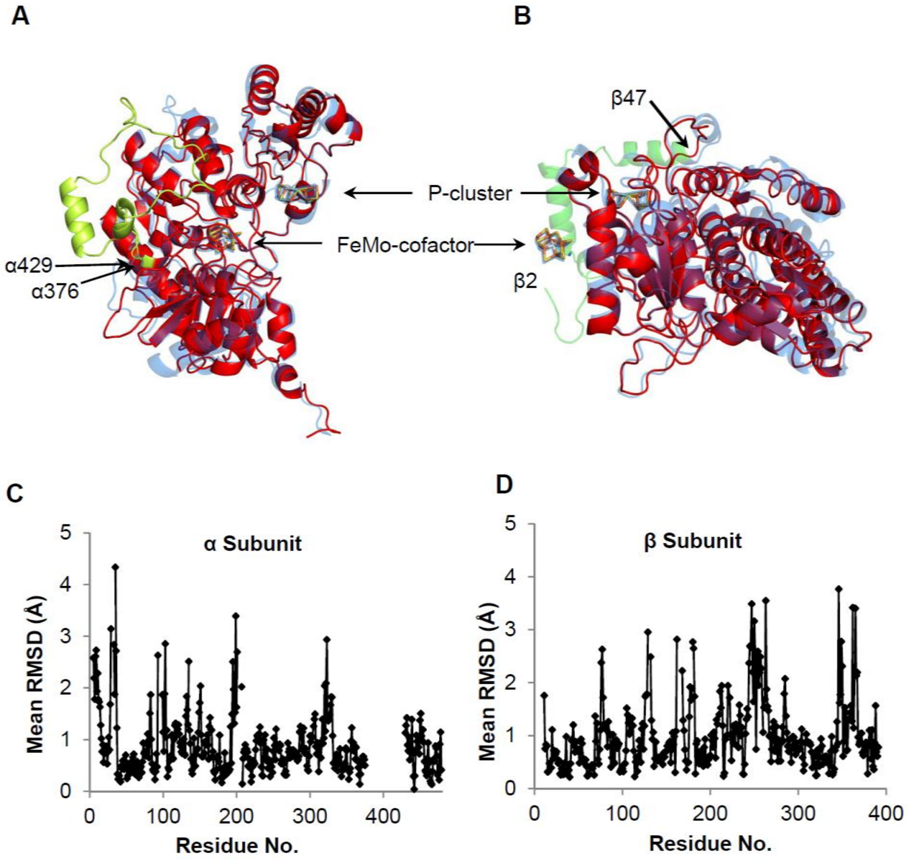

Figure S2. Superposition of the α- and β-subunits of Cp1 and Av1. Cartoon representations of (A) the α-subunits and (B) the β-subunits of Cp1 (red) and Av1 (blue, partially transparent) following superposition of the secondary structures using the program COOT. The α-subunit insertion loop (residues α376 – α429) in Cp1 and the β-subunit deletion (relative to residues β2 – β47 in the Av1 structure) are highlighted in lemon and green, respectively. The two metalloclusters, the FeMo-cofactor and the P-cluster, are highlighted as sticks. The average root-mean-square deviation (RMSD) of least square fitting for two α- and β-subunits are plotted in C and D, respectively, plotted against the residue number of Cp1.

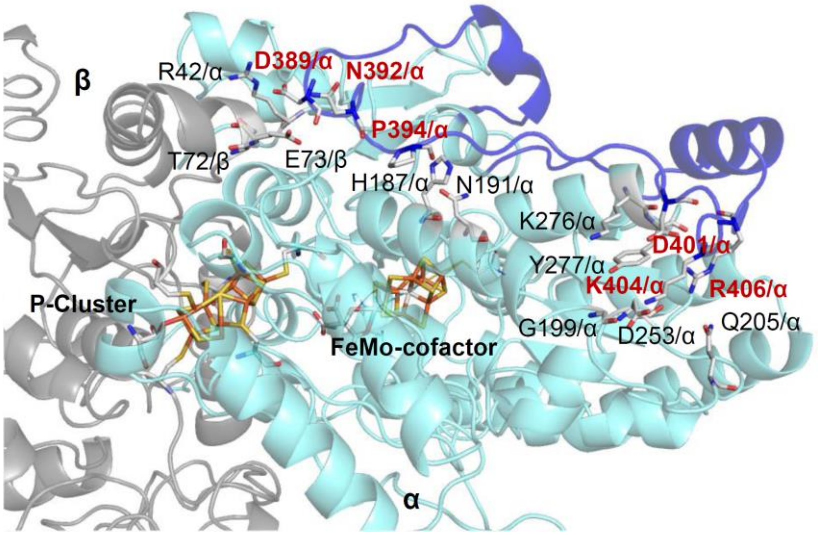

Figure S3. Contacts between the insertion loop and the rest of Cp1. The backbone atoms of the α- and β-subunits are shown as cartoons highlighted in cyan and grey color, respectively, except for the insertion loop (blue). The contacting residues and the two metalloclusters are represented by sticks. Residues in the insertion loop are labeled with red fonts, while other residues are labeled in black. The C atoms are highlighted in light grey color, N in blue, O in red, S in yellow, Fe in orange, and Mo in cyan.

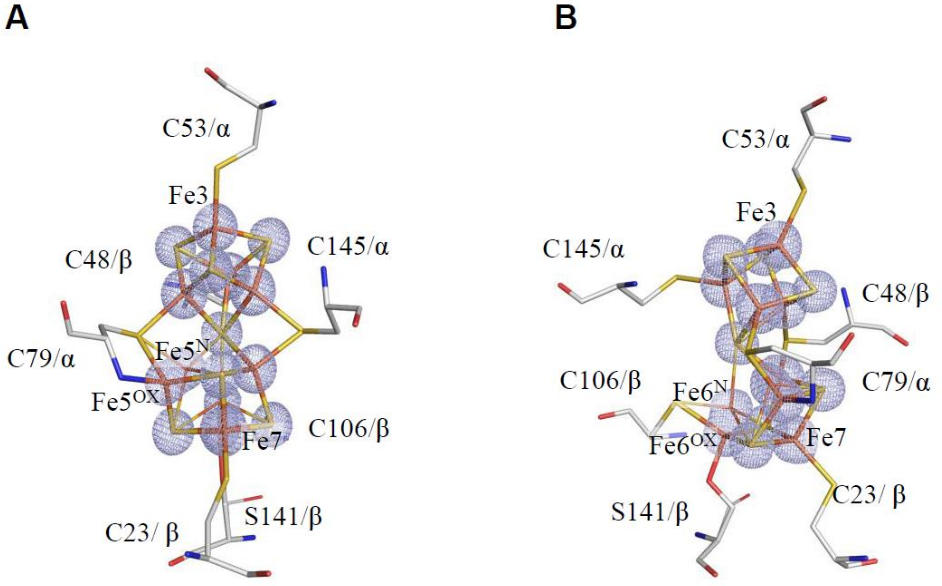

Figure S4. The 2*Fo-Fc* electron density map around the Cp1 P-cluster in two perpendicular views ((A) and (B)). The map is illustrated in light blue color and contoured at 3σ. The P-cluster and coordinated ligand residues are represented by sticks, except for the alterative conformations for Fe5 and Fe6 and the side chain of Ser β141 (corresponding to the reduced P^N state) shown as thin lines. $Fe5^{OX}/Fe6^{OX}$ and $Fe5^N/Fe6^N$ represent the conformations corresponding to the P^{OX} and P^N states, respectively, of the P-cluster. C atoms are highlighted in light grey color, N in blue, O in red, S in yellow, Fe in orange.

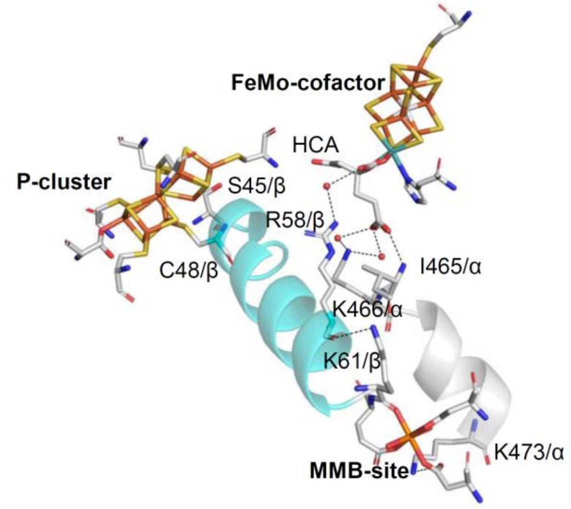

Figure S5. Helices connecting the mononuclear metal binding (MMB)-site and the two metalloclusters, the FeMo-cofactor and P-cluster. The cartoon representations of the two helices from the α- and β-subunit between the MMB-site and the two metalloclusters are depicted in grey and cyan color, respectively. The hydrogen bonding network between water molecules and indicated residues are represented by black dashed lines. C atoms are highlighted in light grey color, N in blue, O in red, S in yellow, Fe in orange, and Mo in cyan. HCA: homocitrate.

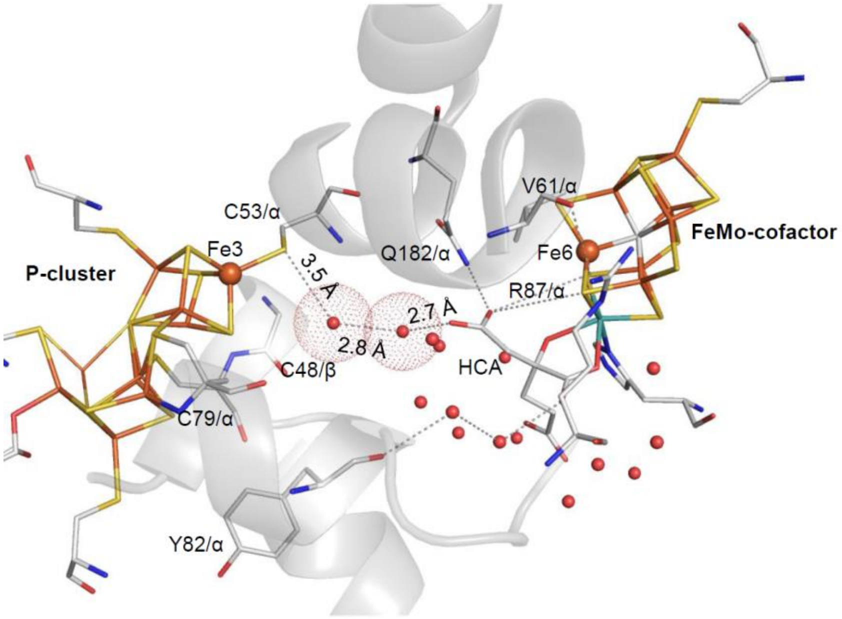

Figure S6. Hydrogen bonding network between the P-cluster and the FeMo-cofactor in Cp1. The four helices from the α- and β-subunits are represented as ribbons in gray color. The P-cluster and the FeMo-cofactor and their coordinated residues are highlighted as sticks, together with residues in the four helices participating in the hydrogen bonding network. Water molecules are shown as red spheres. The two water molecules in the conserved water channel are highlighted with red dotted surfaces. The hydrogen bonding network between water molecules and residues are depicted by black dashed lines. C atoms are shown in light grey color, N in blue, O in red, S in yellow, Fe in orange, and Mo in cyan.
